# Therapeutic Potential of Olfactory Ensheathing Cells and Mesenchymal Stem Cells in Spinal Cord Injuries

**DOI:** 10.1155/2017/3978595

**Published:** 2017-02-16

**Authors:** Zadroga Anna, Jezierska-Woźniak Katarzyna, Czarzasta Joanna, Monika Barczewska, Wojtkiewicz Joanna, Maksymowicz Wojciech

**Affiliations:** ^1^Department of Pathophysiology, Faculty of Medical Sciences, University of Warmia and Mazury in Olsztyn, Olsztyn, Poland; ^2^Department of Neurology and Neurosurgery, Faculty of Medical Sciences, University of Warmia and Mazury in Olsztyn, Olsztyn, Poland; ^3^Laboratory for Regenerative Medicine, Faculty of Medical Sciences, University of Warmia and Mazury, Olsztyn, Poland; ^4^Foundation for the Nerve Cells Regeneration, Olsztyn, Poland

## Abstract

Spinal cord injury (SCI) is a devastating neurological condition that affects individuals worldwide, significantly reducing quality of life, for both patients and their families. In recent years there has been a growing interest in cell therapy potential in the context of spinal cord injuries. The present review aims to discuss and compare the restorative approaches based on the current knowledge, available spinal cord restorative cell therapies, and use of selected cell types. However, treatment options for spinal cord injury are limited, but rehabilitation and experimental technologies have been found to help maintain or improve remaining nerve function in some cases. Mesenchymal stem cells as well as olfactory ensheathing cells seem to show therapeutic impact on damaged spinal cord and might be useful in neuroregeneration. Recent research in animal models and first human trials give patients with spinal cord injuries hope for recovery.

## 1. Introduction

Spinal cord injury (SCI) is debilitating and devastating condition, considered as a major global issue affecting both young and elderly populations. Worldwide, the estimated amount of people living with SCI is about 2.5 million, with more than 130,000 new injuries reported each year. This disorder has a significant impact on life quality and expectancy and is economically burdensome, with considerable costs associated with primary care and loss of income [1]. SCI leads to primary partial or complete loss of motor, sensory and autonomic functions and secondary impairments below the injury level, due to the local spinal cord vasculature damage and the interruption of ascending and descending neural pathways. SCIs are broadly classified into two groups: traumatic and nontraumatic SCI (NTSCI). Patients with NTSCI state minority among the spinal cord population. NTSCI can be a consequence of multiple etiologies including infection, spinal stenosis, vascular impairment, transverse myelitis, syringomyelia, malignant and benign tumors [2]. Traumatic spinal cord injury results from contusion, compression, and stretch of the spinal cord. Trauma related injury is the most prevalent among SCI cases primarily involving road traffic accidents, especially in case of young adults between age group of 15 and 29 years and accidental falls in case of aged people (>65 years) [3]. Nerve cells in the injured segment exhibit necrosis and apoptosis. The necrotic and degenerated tissues are removed by phagocytes and replaced by neuroglial cells, leading to the formation of cystic, melanotic and colloidal lesions at the injured site within 6 weeks after the injury. Then, the physical separation and neural demyelination interrupt the physiological signal transduction pathway, which is marked clinically by a partial or total loss of sensory, motor, urine, and voluntary control of urination and defecation. Physiological neural regeneration is not possible because of injured central nerve axons. Functional reconstruction after spinal cord injury has been a challenging clinical problem [4].

Following surgical interventions that include early spinal decompression and stabilization surgery [5], current treatments used for SCI have mainly neuroprotective or neuroregenerative effect. Neuroprotective therapies focus on impeding or preventing further progression of the secondary injury, whereas neuroregenerative therapies lay emphasis on recovering the lost or impaired functionality by repairing the broken neuronal circuitry of the spinal cord [6, 7]. Preclinical research has revealed that many elements of the secondary injury cascade occur over a prolonged period of time after injury, providing an opportunity for neuroprotective exogenous treatments to be effective if applied within this time period [8, 9]. The evaluation of patient's condition is based on classification of spinal cord injury severity using American Spinal Injury Association (ASIA) Impairment Scale. The main categories of the Impairment Scale are as follows: (A) total lack of sensory and motor function below level of injury, (B) some sensation below level of injury, (C) >50% of muscles below level of injury cannot move against gravity, (D) >50% of muscles below level of injury can move against gravity, and (E) all neurologic function has returned. In general, the effectivity of therapy in spinal cord injuries is established using ASIA scale [10].

Due to the complex nature of injury, several therapeutic strategies are combined to treat various aspects of the trauma. Neuroprotection pertains to the preservation of the spared neurons and their processes immediately following the injury, since the events that occur during the secondary injury or expansion phase harm the spared, once fully functional neurons. Neuroregeneration aims to modulate the lesion site environment to promote axonal regrowth by removing inhibitory growth substances and providing a growth supportive environment. Consequently, intraspinal transplants enrich the lesion site by replacing lost cells with new neurons and/or glial cells to create and restore functional connections or provide a more permissible medium for regenerating axons. Neurorehabilitation in a form of exercise/physical training has demonstrated beneficial effects at the cellular and molecular levels and may translate into recovery of function [11].

**Figure 1:**
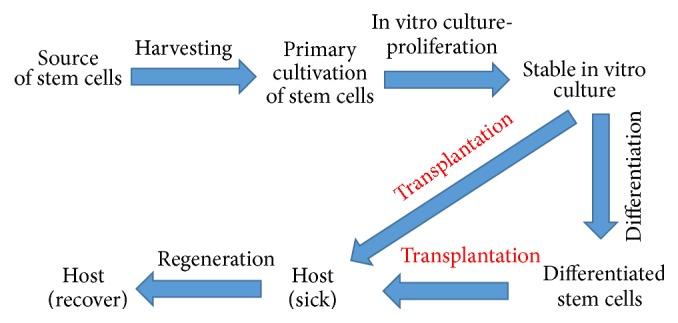
Scheme: therapy of stem cells.

**Figure 2:**
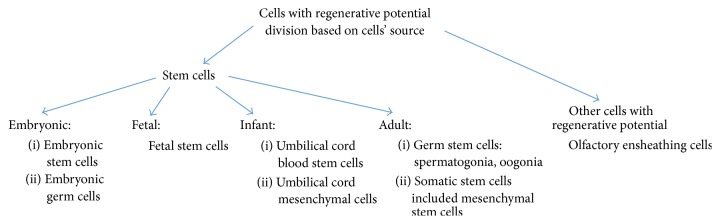
Classification of cells with regenerative potential.

So far, a few approaches have been performed to increase the rate of improvement in nerve regeneration applications. One of them is a stem cell-based strategy, which is a very promising therapy for repairing the SCI (a general scheme of stem cell-based therapy is shown in Figure 1). Various types of stem cells from different sources were tested in the regeneration of damaged neural cells. Different cell sources for transplantation might be required for optimal clinical improvement, depending on type of the pathophysiology of the injury [12]. Grafting of somatic cells and tissues such as olfactory ensheathing cells (OECs), Schwann cells, fetal tissues, and peripheral nerves has made the SCI microenvironment more favorable for neural regeneration. On the other hand, neural progenitor/stem cells, embryonic stem cells, induced pluripotent stem cells, mesenchymal stem cells (MSCs), fibroblast-derived stem cells, and others are all exploited for their pluripotent differentiation ability to replace neuronal lineage cells, enhance axonal regeneration, and restore interneuron communications [13]. Following our literature studies we concluded that optimal adjuvant therapies for patients who suffered from SCI should have three main properties: (1) neurotrophic abilities to stimulate axonal growth from regular, existing cells; (2) immunomodulation to stop cell death; and (3) the competence to replace injured cells. The source of cells with regenerative potential should be discussed before the choice for particular therapy (classification based on the source of those cells is shown in Figure 2). This review is focused on therapies applying olfactory ensheathing cells, which are proved to act as stimulating axonal growth factors [14] and mesoderm derived mesenchymal stem cells (mainly derived from bone marrow) and their implantation in patients with SCI which are believed to occur through regulation of the immune system, leading to decreased cell death [15].

## 2. Olfactory Ensheathing Cells and Mesenchymal Stem Cells as Promising/Potential Candidates for Therapy of Patients with Spinal Cord Injuries

### 2.1. Olfactory Ensheathing Cells

#### 2.1.1. Characteristic and Research

OECs are unique cells that are responsible for the expression of various neurotrophic factors, which are important for the extension and guidance of axons. They are a population of glial cells residing both in the peripheral and central nervous systems. Together with their accompanying envelope of olfactory nerve fibroblasts (ONFs), they enfold the bundles of olfactory nerve fibers in their path from the nasal mucosa to make synaptic connections in the olfactory bulb [16–19]. OECs share properties with astrocytes and Schwann cells [20]. The key ability of OECs from the perspective of neural regeneration is their migration from peripheral to the central nervous system. As a consequence the enhancement of axonal extension after injury is possible and can help neural regeneration. During embryonic olfactory system development, neural cell adhesion molecule (NCAM) and L1/neuron-glia cell adhesion molecule (L1/Ng-CAM) in the membrane of OECs enable the olfactory axons to take the glial cell surfaces as a substratum on which they grow, and the secreted laminin and nexin from OECs provide other adhesive substrates for the olfactory axons as the neuron-promoting agents [21]. OECs migrate into the injury site, enhance the axon growth due to permissive OEC environment during neural regeneration [22]. Because of mentioned OECs properties for continuous regeneration and their stimulation of axonal growth, an increasing number of studies have attempted to transplant OECs into injured spinal cord for potential therapeutic use in neural regeneration.

In the last decade, the research on animals have shown that transplantation of OECs and ONFs cultured from the olfactory bulb mediate regeneration and functional reconnection of severed axons in spinal cord injuries [23–28]. In particular, Barbour and coauthors have demonstrated significant increase of neuronal cell survival after olfactory ensheathing cell transplantation in the rat model. Additionally, in the mouse model, Witheford et al. observed the ability of OECs to secrete adhesion molecule L1. Subsequently, those cells have stimulated axonal growth through the L1 activity. In another research, the functioning axons were identified at the injury site after OECs transplantation to the injured rat spinal cord [26]. Similarly, functional challenge was observed: three of nine rats with OECs treatment after SCI start to move their hind legs while 12 controls did not show any improvement.

To date, preliminary clinical trials seem to be inconsistent, but multiple small studies demonstrated benefits and safety of using OECs in humans. For example, Lima et al. [29] observed 55% effectiveness in ASIA grade improvement. During this study, 11 of 20 patients noticed an improvement after such treatment, including five patients who recovered voluntary bowel control and one who recovered bladder control. In another clinical trial only one of six patients with chronic SCI had any neurologic improvement [30]. The results obtained by Wu et al. [31] demonstrated safety of therapy with fetal olfactory ensheathing glia transplantation in patients with SCI. In this clinical trial, the olfactory bulb was harvested from 16-week-old fetuses following strict ethical guidelines. All eleven patients had no complication of neurological conditions. Three of five patients with low cervical cord injury had improvement of muscle strength below the level of injury: in patients with thoracic injury, the motor improvement was not observed, even though five of six patients had improved sensation to gentle touch and pinprick [31].

Recently, Tabakow et al. [32] have reported clinical trials using cells taken from the olfactory bulb and olfactory mucosa in human SCI patients. In this clinical trial, six patients received autologous OEC transplantation in a Phase I clinical trial, of whom three patients showed signs of improvement of SCI, and two demonstrated improvement according to ASIA scale (ASIA (A) to (C) and (B)). Researchers concluded after one year observations that the obtaining, culture and intraspinal transplantation of autologous OECs is safe. Furthermore, they considered that transplantation of OECs was the main factor contributing for the neurological improvements in the three patients with transplants. Study of this research group suggested the improvement is a consequence of transplanted OECs which may mediate some restitution of efferent and afferent long white matter tracts in three treated patients [32].

The latest results indicate that the transplantation of autologous bulbar cells in 38-year-old patient has been successful. Using MRI, it was found that grafts had braided the left side of the spinal cord, where the majority of these nerve grafts were implanted, and neurophysiological examinations confirmed the restitution of the integrity of the corticospinal tracts and the voluntary character of recorded muscle contractions [33].

### 2.2. Mesenchymal Stem Cells

#### 2.2.1. Characteristic and Research

Mesenchymal stem cells are an umbrella term for adult stem cells originating from mesoderm. These cells could be harvested from multiple tissues, such as bone marrow, umbilical cord, adipose and pancreatic tissue [34]. The bone marrow-derived MSCs (BM-MSCs; the best known source of MSCs) belong to the multipotent somatic cells. In the process of cell isolation and in vitro culture, MSCs must be separated from hematopoietic stem cells before implantation. The benefit of allogenic transplantation relies on the lack of the class II major histocompatibility complex expression in those cells [15]. BM-MSCs are an innovative therapeutic tool in the treatment of a number of diseases through their neuroprotective and paracrine ability [35]. In the last decade, research focused on cell activity demonstrated significant neurotrophic properties of BM-MSCs. Those cells secrete nerve growth factor and neurotrophin-3, which support axonal growth [36]. In addition, MSCs offer the advantages of (1) being an easily obtainable source; (2) possessing the ability for expansion in vitro; (3) lacking a requirement for immunosuppressive therapy to prevent rejections; and (4) having a reduced risk of malignant transformation [37]. The recent experiment of [37] research team demonstrated induction of MSCs to secrete neurotrophic factors (MSC-NTF cells) and implementing the cells in clinic for therapy of patients with amyotrophic lateral sclerosis. In the procedure, using a medium-based differentiation process, they have induced MSCs to become MSC-NTF cells, with markedly enhanced secretion of NTFs such as GDNF, brain-derived NTF, vascular endothelial growth factor (VEGF), and hepatocyte growth factor (HGF) [38].

The motor function recovery through an axonal regeneration after MSCs transplantation to the spinal cord was confirmed in animal studies. A lot of cytokines and growth factors such as neurotrophins, colony-stimulating factor (SCF), interleukins, stem cell factor (SCF), NGF, BDNF, HGF, and VEGF are expressed in MSCs. In rodent, the effect of MSCs transplantation was depending on time of grafting cells: the positive impact was noticed when cells were transplanted one week after injury (BBB scores), while transplantation of MSCs four months after SCI had no effect on examined parameters (BBB scores). It is generally proposed that this window spans between three days and three weeks after SCI [39]. In another rat model of SCI, increase in axonal growth factor levels as demonstrated in rats treated with MSCs compared to control rats. Additionally, new axons were oriented in the proper way and inhibition mechanism of T-cell activation has proven to preserve host neurons and myelin in rodents treated with MSCs compared with controls [40].

The hypothetical ability of MSCs to replace cells of the central nervous system has not been proven, even though some studies showed their ability to at least temporarily function as replacement cells. Firstly, Hofstetter et al. [41] observed that MSCs expressed neural markers and were tightly associated with immature astrocytes five weeks after SCI. However, the research team concluded that these cells could not perform the astrocytes function. In another animal study, 85% of MSCs transplanted into injured spinal cords of rats expressed neural cell markers two weeks after procedure. However, twelve weeks after surgery, only 10% of MSCs stayed positive for neural markers [42].

Some clinical trials which showed beneficial results and safety of using MSCs in humans have been conducted. However, the effects of these trials which certainly are very complex and require the highest precision of neurosurgeons are still ambiguous. In the research of Bhanot et al. [43] 13 patients with chronic SCI (class A of ASIA scale) were treated with laminectomy, followed by implantation of autologous MSCs into the spinal lesion. Three of these patients demonstrated some reaction: one of them had a slight improvement in motor function and two of them expressed pinprick sensation below the level of injuries. Another research team has been implemented autologous MSCs in patients with acute and subacute SCI [44]. Approximately 30% of these patients had at least one neurological improvement on ASIA scale, even though it is hard to estimate whether it was not the effect of the natural process of acute SCI [44].

## 3. Perspectives

### 3.1. Possibilities of OECs and MSCs Injection to Injured Spinal Cord

Considering the above mentioned specific characteristics of OECs and MSCs, these cells may influence the outcomes of therapeutic strategy for spinal cord injuries. Previously described preclinical and clinical studies also confirm their positive effective impact on the toxic environment in the injured spinal cord. However, there are no clear guidelines for a method of cell delivery to the damaged area of the spinal cord. It is necessary to conduct further research and establish an effective injection system. Scientists are looking to design a microinjection system, which will be mounted to the patient's spine for optimal stability and electronically controlled administration of OECs and MSCs to the injured spinal cord. The injection will be immobilized relative to the spine with percutaneous mounts attached to vertebral pedicles flanking the injection site. The spine mounts will allow the injection system to move with the patient during ventilation and in the event of inadvertent patient movement. The stabilized platform also would allow for accurate landmark-based targeting with the adjustable microinjector attached to the platform. This injection system will use an outer rigid cannula for accurate targeting and an inner flexible or floating cannula for cell delivery. Hopefully, these innovations will reduce the procedural risks associated with direct intraspinal cord injection and improve targeting capability.

## 4. Conclusion

A presented review attempted to discuss and compare the restorative approaches based on the current knowledge about available spinal cord restorative cell therapies and use of selected cell types. We believe that there is a strong need to help people suffering from spinal cord injuries to recover and come back to normal life. SCIs have a significant impact on life quality and expectancy and are economically burdensome, with considerable costs associated with primary care and loss of income. Treatment options for spinal cord injuries are limited, but rehabilitation and experimental technologies have been found to help maintain or improve remaining nerve function in some cases. Regenerative abilities of OECs and MSCs are still not enough understood and therefore the effective treatment of SCI has not been developed yet. However, recent development of stem cell approaches has highlighted their usefulness in treatment and give hope for patients with spinal cord injuries. A novel strategy that combines several disciplines such as neurology, neurosurgery, bioengineering, and stem cell therapy, which focus on therapeutic treatment of children and adults with spinal cord injuries, should be quickly implemented to improve patients' quality of life.
