# Corrigendum to “Therapeutic Potential of Olfactory Ensheathing Cells and Mesenchymal Stem Cells in Spinal Cord Injuries”

**DOI:** 10.1155/2017/9438310

**Published:** 2017-05-31

**Authors:** Anna Zadroga, Katarzyna Jezierska-Woźniak, Joanna Czarzasta, Monika Barczewska, Joanna Wojtkiewicz, Wojciech Maksymowicz

**Affiliations:** ^1^Department of Pathophysiology, Faculty of Medical Sciences, University of Warmia and Mazury in Olsztyn, Olsztyn, Poland; ^2^Department of Neurology and Neurosurgery, Faculty of Medical Sciences, University of Warmia and Mazury in Olsztyn, Olsztyn, Poland; ^3^Laboratory for Regenerative Medicine, Faculty of Medical Sciences, University of Warmia and Mazury, Olsztyn, Poland; ^4^Foundation for the Nerve Cells Regeneration, Olsztyn, Poland

In the article titled “Therapeutic Potential of Olfactory Ensheathing Cells and Mesenchymal Stem Cells in Spinal Cord Injuries” [1], the first and last names of five of the authors were reversed as Zadroga Anna, Jezierska-Woźniak Katarzyna, Czarzasta Joanna, Wojtkiewicz Joanna, and Maksymowicz Wojciech. The authors' names should have been written as Anna Zadroga, Katarzyna Jezierska-Woźniak, Joanna Czarzasta, Joanna Wojtkiewicz, and Wojciech Maksymowicz. The revised authors' list is shown above.
